# Neurotrophins expression in brain regions of an anorexia nervosa mouse model, biomarkers for diagnostic and prognostic

**DOI:** 10.1192/j.eurpsy.2025.1537

**Published:** 2025-08-26

**Authors:** N. Ramoz, J. Cao, V. Tolle, P. Gorwood, O. Viltart

**Affiliations:** 1 U1266, INSERM; 2 CMME, GHU PARIS Psychiatrie & Neurosciences, Paris; 3 Université Lille, Lille, France

## Abstract

**Introduction:**

Anorexia nervosa (AN) is a complex mental disorder characterized by a voluntary food restriction and excessive physical activity resulting in dramatic weight loss. Neurotrophins are growth factors that play s role in the proliferation, survival and differentiation of cells in the brain. Alterations in neurotrophin factors, including the brain-derived neurotrophic factor (BDNF), have been found in patients wuth AN. Variants of genes encoding various neurotrophins and their receptors have been associated with eating disorders or with disease outcome or prognosis compared with controls. In animal, BDNF negatively modulates the central control of food intake and its injection into rodents induces weight loss and anorexia. Its receptor, the p75 neurotrophin receptor was also able to regulate the energy balance, as observed in mice lacking the p75NTR, which decreased their feeding. Thus, several neurotrophins and their receptor may have a metabolic effect, making them good candidates for biomarkers in AN for diagnostic and prognostic purposes.

**Objectives:**

Our work measured the levels of expression of neurotrophins and their receptors in specific brain areas, taking advantage of the mouse AN-like model in food-restricted and refeed animals.

**Methods:**

The AN-like model combining a chronic food restriction (50%) for 15 days followed by an *ad libitum* refeeding period of one week was used. Female mice have access to a running wheel to create a metabolic environment similar to that of patients suffering from AN during restriction and recovery after hospitalization. The mRNA levels of Bdnf, TrkB, Ngf, NtF3 and Ntf5 were measured in brain regions (prefrontal cortex, hippocampus, hypothalamus and dorsal striatum) using quantitative PCR in the different groups of mice (ad libitum, ad libitum with wheel, food restriction and food restriction with wheel). Statistical analysis will compare levels using one-way or two-way ANOVAs depending on the animal group or brain region.

**Results:**

A significant decrease of the expression of *Bdnf*, *Ngf* and *Ntf5* genes was observed between *ad libitum* and food restriction groups in the dorsal striatum. The transcript level for *Bdnf* was also decreased in the prefrontal cortex in the food restriction groups compared to the *ad libitum* groups. In contrast, TrkB transcript was increased in in food restriction groups compared to ad libitum groups, in the prefrontal cortex. We expect significant differences in gene expression in the other brain regions of interest for the food restricted animals compared to the *ad libitum* animals. The levels of neurotrophic genes are ongoing for the refeeding animals.

**Conclusions:**

Neurotrophins could represent potential biomarkers of AN for the diagnosis and prognosis in the evolution to weight recovery and will thus allow a better understanding of the aetiology of AN.

This work was supported by Fédération Recherche sur le Cerveau.

**Disclosure of Interest:**

None Declared

